# Differences in Cardiovascular, Biochemical and Nutritional Parameters Between High- and Low-Altitude Winter Sports Athletes

**DOI:** 10.3390/nu17233665

**Published:** 2025-11-24

**Authors:** Maria Jose Jimenez-Casquet, Javier Conde-Pipó, Josep A. Tur, Miguel Mariscal-Arcas

**Affiliations:** 1 Health Science and Nutrition Research (HSNR-CTS1118), Department of Nutrition and Food Science, School of Pharmacy, University of Granada, 18071 Granada, Spain; mariajosejcf@correo.ugr.es; 2 Department of Health Sciences, Faculty of Health Sciences, University of Jaén, 23071 Jaén, Spain; 3 Research Group on Community Nutrition and Oxidative Stress, University of Balearic Islands-IUNICS & Laboratory of Physical Activity Sciences, 07122 Palma de Mallorca, Spain; pep.tur@uib.es; 4 Centro de Investigación Biomédica en Red Fisiopatología de la Obesidad y la Nutrición (CIBEROBN), Institute of Health Carlos III, 28029 Madrid, Spain; 5 Health Research Institute of the Balearic Islands, 07120 Palma de Mallorca, Spain; 6 Instituto de Investigación Biosanitaria de Granada (ibs.GRANADA), 18016 Granada, Spain

**Keywords:** ECG, training high, hypoxia, Mediterranean diet, alpine skiing

## Abstract

**Background/Objectives**: High-altitude hypoxia may affect ECG readings, but it is unclear whether the “live-low–train-high” approach prevents these changes in winter sports athletes. **Methods**: This cross-sectional study assessed cardiovascular parameters in 102 winter-sport athletes (mean age 20 ± 4 y; 57% women), divided by training altitude into a high-altitude (HA) group (2500–3300 m, n = 70; skiers/snowboarders) and a low-altitude (LA) group (738 m, n = 32; ice hockey/figure skaters). Mid-season assessments included resting ECG, blood pressure, blood biochemistry, and three 24 h dietary recalls. **Results**: All ECG parameters were physiological, and no significant differences (*p* < 0.05) were observed in heart rate, PR interval, or QTc between groups. However, HA group exhibited higher systolic blood pressure and a short QT interval. Lactate was significantly higher in HA (*p* = 0.028). The HA diet contained more saturated fat (*p* < 0.001), cholesterol (*p* = 0.013), magnesium (*p* = 0.003) and potassium (*p* = 0.001), whereas LA athletes consumed more glucose (*p* = 0.024). In HA, total energy expenditure correlated positively (*p* ≥ 0.05) with QRS (ρ = 0.52) and QT (ρ = 0.56), while heart rate correlated inversely with vitamin D (ρ = −0.59). In LA, QTc showed strong inverse correlations with zinc (ρ = −0.62) and selenium (ρ = −0.85). **Conclusions**: This finding suggests that intermittent high-altitude training did not alter ECG patterns when nutrient intake was adequate. High lactate level and specific nutrient correlations point to a residual physiological load and a modulatory role of electrolytes, B-vitamins, and vitamin D on cardiac repolarisation.

## 1. Introduction

Sudden cardiac death (SCD) is the primary cause of mortality among athletes, with many of these cases being attributed to undetected cardiovascular conditions [1,2]. Therefore, identifying conditions that predispose individuals, especially young or elite athletes, to SCD is crucial, and this can be achieved by detecting electrical abnormalities through a resting electrocardiogram (ECG). Due to its non-invasive nature and high sensitivity, the ECG can distinguish between physiological adaptations resulting from chronic training and pathological electrical alterations that could potentially trigger sudden death [2].

In recent years, international guidelines have been published to standardise ECG interpretation in athletes, help prevent undesirable cardiac events, and minimise false positives [1,2]. However, as most recommendations are based on populations living at sea level, it is unclear whether they are applicable to individuals training or living in hypobaric hypoxia [3]. High altitude, typically above 2500 m, is characterized by reduced atmospheric oxygen pressure (PO_2_), resulting in lower maximal oxygen uptake ($\dot{V}$O_2_max), arterial oxygen pressure (PaO_2_), and arterial oxygen saturation (SaO_2_). Hypobaric hypoxia triggers multiple cardiovascular, neurological, metabolic, and respiratory adaptations that may increase cardiovascular risk, often resulting in electrocardiogram (ECG) changes [3,4].

Common ECG changes at high altitude include right axis deviation, increased P-wave amplitude, incomplete right bundle branch block, and T-wave inversion, reflecting right ventricular strain and physiological adaptation to hypoxic stress [4,5]. Nevertheless, reports of altitude-specific ECG patterns remain limited, outdated, and primarily based on case reports or small cohort studies [6]. Consequently, the specific ECGs modifications during acute exercise in hypoxia remain poorly characterised, and it can sometimes be challenging to differentiate pathological findings from the physiological adaptations typically observed in the so-called athlete’s heart [7]. Coustet et al. [5] reported that no significant arrhythmias or QRS axis changes were observed in healthy, trained individuals during acute hypoxic exposure. However, they did observe a generalised decrease in P, QRS and T wave amplitudes, suggesting that these electrical changes represent physiological adaptation to hypoxic stress rather than underlying pathology.

In this context, recent studies indicated that athletes are not at a higher risk of ventricular arrhythmias when training or competing at high altitudes [8,9]. Nevertheless, for sea-level athletes, the optimal strategy to maximise the benefits and minimise the risks of altitude training remains under debate, with the “living high, training low” (LH-TL) approach currently considered the most effective [9,10].

Winter sports provide an ideal model for studying this phenomenon, as snow-based disciplines such as alpine skiing, freestyle, and snowboarding are typically trained at resorts located between 2000 and 3000 m above sea level [11]. However, despite training at high altitude, alpine skiers often adopt a “live low, train high” (LL-TH) strategy, residing at lower elevations but spending several days or even weeks at high altitude during training periods, competitions, or school breaks. Although these exposures to altitude are relatively brief, the LL-TH approach in skiers is known to induce physiological adaptations to hypoxia, including morpho-functional muscle changes, increased muscle buffering capacity, higher myoglobin content, enhanced mitochondrial function, and greater capillarization of working skeletal muscles [12]. However, its impact on ECG patterns remains less clearly understood and existing studies are notably scarce [13,14,15,16].

On the other hand, the relationship between nutrition and cardiac electrophysiology is supported by well-established mechanisms [17,18]. Specific electrolytes (K^+^, Mg^2+^, Ca^2+^) and water-soluble vitamins play a direct role in stabilizing membrane potential and regulating the duration of repolarization. Several studies in the general population indicated that decreased potassium levels or hypomagnesemia can prolong the QTc interval, thereby increasing the risk of torsades de pointes [19,20]. In individuals with long QT syndrome, potassium supplementation shortened the QTc or, at least, reduced repolarization dispersion [21]. However, the effectiveness of potassium elevation in reducing the QT interval remains controversial and may not provide substantial antiarrhythmic [19]. The dietary influence extends beyond electrolytes; adequate intake of antioxidants (vitamin E, polyphenols), polyunsaturated fatty acids, and vitamin D were associated with modulation of inflammatory and autonomic processes that, in turn, influenced myocardial excitability [17]. Therefore, nutritional factors could modulate ECG variations resulting from altitude exposure.

In conditions of hypobaric hypoxia, additional factors contribute to physiological stress: (i) increased diuresis and ventilation, leading to fluid and electrolyte losses; (ii) reduced thirst sensation; and (iii) appetite suppression, resulting in lower overall caloric intake [22,23]. These effects can lead to states of low energy availability and micronutrient deficiencies—particularly in iron, calcium, and vitamin D—which may disproportionately affect female athletes [24,25]. While some studies have begun to explore the impact of nutrition on ECG patterns in athletes exposed to altitude, the evidence remains limited, underscoring the need for further research to better understand these interactions [26].

It might be possible to hypothesise that exposure to altitude is beneficial for health and athletic performance with a diet carefully adapted to the demands of altitude, helping athletes maintain electrical and metabolic stability of the heart throughout the competitive season. This study aimed to assess the impact of altitude exposure on ECG patterns in athletes, focusing on the direct effects of hypoxia and the potential modulatory role of nutrition. By comparing ECG parameters between athletes training at high and moderate altitudes, and examining the influence of key nutrients (potassium, magnesium, vitamin D), we sought to clarify how nutritional status may exacerbate or mitigate hypoxia-induced ECG changes, particularly in female athletes. This integrative approach will provide a comprehensive perspective on how altitude exposure and nutritional status interact to influence cardiac electrophysiology in winter sports athletes.

## 2. Materials and Methods

### 2.1. Study Design and Participants

This cross-sectional, descriptive, and comparative study was conducted in collaboration with the Andalusian Federation of Winter Sports and the Department of Nutrition and Food Science at the University of Granada, Spain. The study included 102 athletes aged 20.1 years (SD: 4.2) competing in various winter sports: alpine skiing (53.5%), freestyle skiing/snowboarding (15.8%), ice hockey (14.8%), and figure skating (15.8%). Based on their primary sport, participants were divided into two groups: the high-altitude group (HA), consisting of alpine skiing, freestyle skiing, and snowboarding athletes who primarily trained in Sierra Nevada (Granada, Spain) at altitudes ranging from 2500 to 3300 m, and the low-altitude group (LA), comprising ice hockey and figure skating athletes who trained in Granada city (Spain) at a stable altitude of 738 m.

Inclusion criteria consisted of being a registered athlete in the Andalusian Winter Sports Federation, engaging in at least 10 h of training per week, and being at least 10 years old. Exclusion criteria encompassed any cardiovascular, thyroid, or chronic medical conditions that could impact the study variables. Data collection was performed during the competition season.

The study was conducted following the Declaration of Helsinki guidelines and was approved by the Research Ethics Committee of the University of Granada (ref. 1162/CEIH/2020). Prior to participating in the study, all participants were informed of the objectives of the research and provided their written informed consent. For athletes under 18 years of age, informed consent was obtained from their parents or legal guardians.

### 2.2. Procedures

#### 2.2.1. Electrocardiographic Assessment, Blood Pressure, and Blood Analysis

Resting ECG measurements were obtained using a Mortara Eli 150c electrocardiograph (Mortara Instrument, Milwaukee, WI, USA). The parameters assessed included heart rate, PR interval, QRS duration, QT and QTc intervals. Participants were positioned supine and rested for 10 min prior to the recording to ensure baseline cardiac activity [1].

Blood pressure was measured using a validated Omron HEM-705CP semiautomatic oscillometer (OMRON Corporation, Kyoto, Japan). Measurements were taken in a seated position with a 5-min rest interval between readings. The mean of both measurements was used for further analysis.

Venous blood samples were drawn from the antecubital vein in the morning after a minimum 8-h fast. Samples were immediately processed in local laboratories using Abbott Architect c16000 equipment (Abbott Diagnostics, 150 S. Saunders Rd., Lake Forest, IL, USA). Parameters analysed included hematological profile, inflammatory markers, coagulation factors, and lipid profile.

#### 2.2.2. Anthropometric Assessment

Trained ISAK-certified personnel conducted all anthropometric assessments, following international standards [27]. Height was measured using a wall-mounted stadiometer (Seca 214, Seca gmbh & co., Hammer Steindamm, Hamburg, Germany) and weight using a Tanita BC-418 scale (Tanita Europe, Amsterdam, The Netherlands), with participants barefoot and wearing light clothing. A standard weight adjustment of 0.6 kg was applied for clothing and participants were advised to avoid high intensity exercise the day before the measurements were taken, and to not perform any training or stretching sessions on the day of the assessment [27,28].

Body mass index (BMI) was calculated using the formula: weight (kg)/height (m^2^). Skinfold thickness (bicipital, tricipital, subscapular, suprailiac, abdominal, mid-leg, and thigh) was measured using a Holtain plicometer (Holtain Ltd., Crymych, UK). Faulkner’s equation was applied to estimate body fat percentage. Each measurement was performed in triplicate, and the mean value was recorded for analysis [29]. All anthropometric, electrocardiographic, and biochemical measurements were performed on the same day during a single standardized testing session to ensure temporal consistency.

#### 2.2.3. Energy Expenditure Estimation and Dietary Assessment

Basal metabolic rate (BMR) was calculated using the Harris-Benedict equation [30]. Total energy expenditure (TEE) was estimated following the FAO/WHO/UNU guidelines, incorporating BMR, physical activity level (PAL), and thermic effect of food [31]. Dietary intake was assessed using a 3-day 24-h recall (R24h) questionnaire [32], conducted by trained interviewers. Participants provided detailed accounts of all foods and beverages consumed over three non-consecutive days, including one weekend day. Nutrient intake data were analysed using the DIAL program (2015 Alce Ingeniería, Las Rozas, Madrid, Spain) and the AUSNUT 2011-13 food database which was selected because it provides a comprehensive nutrient composition, including micronutrients not fully covered by Spanish databases. Reliability of the dietary data was evaluated using the Goldberg cut-off criteria [33,34]. Although biochemical analyses were performed, they were not intended as dietary biomarkers to validate nutrient intake. The 24-h recalls were therefore used as the primary method for dietary assessment, and future studies should include specific biochemical markers (e.g., 25(OH)D, plasma carotenoids) to objectively validate nutrient intake estimates.

#### 2.2.4. Statistical Analysis

Statistical analysis was performed with R statistical software (version 4.1.2; R Core Team, Vienna, Austria). Normality was examined with the Kolmogorov–Smirnov test using the Lilliefors correction, and homoscedasticity with Levene’s test. Means ± standard deviations (SD) were reported for descriptive purposes. Group comparisons of continuous variables were carried out with the non-parametric Mann–Whitney U test; effect sizes were expressed as the rank-biserial correlation. Categorical variables (e.g., sex) were compared between groups using the chi-square test. Bivariate associations were evaluated with Spearman’s rho correlation coefficient. All *p*-values are two-tailed, and statistical significance was set at α = 0.05 (95% confidence level). Correlation analyses were exploratory and intended to identify potential associations between nutrient intake and cardiovascular parameters. Therefore, no formal correction for multiple testing was applied.

## 3. Results

A total of 102 athletes were included in the analysis, classified into high-altitude (HA; n = 70) and low-altitude (LA; n = 32) groups based on their primary sport. No significant differences were observed in age, height, weight, or BMI between groups (Table 1). Although skinfold thickness and body fat percentage were slightly higher in the LA group, these differences did not reach statistical significance.

As shown in Table 2, resting ECG parameters and blood pressure values were comparable between groups. Heart rate, PR interval, QRS duration, QT, and QTc intervals, as well as systolic and diastolic blood pressure, did not differ significantly. All values remained within physiological ranges.

Regarding blood biochemistry (Table 3), only lactate levels were significantly higher in the HA group compared to LA (1.7 ± 0.5 vs. 1.3 ± 0.5 mg/dL, *p* = 0.028, r = 0.21). No significant differences were found for hemoglobin, hematocrit, cholesterol, glucose, or electrolyte levels.

Nutrient intake analysis (Table 4 and Table 5) revealed a higher intake of glucose (*p* = 0.024, r = −0.51) in the LA group. The HA group had significantly higher intake of and cholesterol (*p* = 0.013, r = 0.52), saturated fat (*p* < 0.001, r = 1.03), magnesium (*p* = 0.003, r = 0.30), potassium (*p* = 0.001, r = 0.33), manganese (*p* < 0.001, r = 0.37), phosphorus (*p* = 0.044, r = 0.20), iodine (*p* = 0.032, r = 0.21), fluorine (*p* < 0.001, r = 0.38), folic acid (*p* = 0.018, r = 0.24), vitamin E (*p* = 0.036, r = 0.21), and β-carotene (*p* = 0.027, r = 0.22).

**Figure 1:**
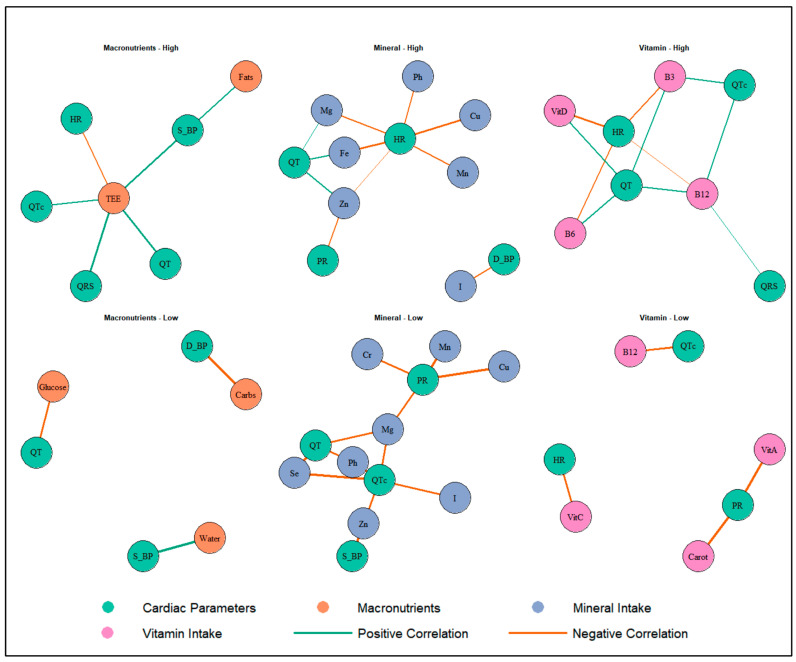
Associations Between Nutritional Intake and Cardiac Parameters in Athletes Exposed to Altitude. HR: heart rate; PR: PR interval; QRS: QRS duration; QT: QT interval; QTc: corrected QT interval; D_BP: = diastolic blood pressure; S_BP: systolic blood pressure; TEE: total energy expenditure; Water: total water intake; Carbs: carbohydrates; Fe: Iron; I: iodine; Zn: zinc; Mg: magnesium; Mn: manganese; Cu: copper; Cr: chromium; B3: niacin; B6: vitamin B6; B12: vitamin B12; VitC: vitamin C; VitA: vitamin A; VitD: vitamin D; Carot: beta-carotene.

Correlation analysis (Table 6 and Table 7; Figure 1) suggests distinct patterns by altitude group. In the HA group, total energy expenditure (TEE) was positively associated with QRS duration (rho = 0.52, *p* < 0.001), QT interval (rho = 0.56, *p* < 0.001), QTc interval (rho = 0.35, *p* < 0.05), and systolic pressure (rho = 0.55, *p* < 0.01). QT interval showed positive associations with vitamin D (rho = 0.42, *p* < 0.01), vitamin B6 (rho = 0.45, *p* < 0.01), and niacin (rho = 0.47, *p* < 0.01), while QTc was associated with vitamin B12 (rho = 0.39, *p* < 0.05).

Heart rate was inversely associated with iron (rho = −0.59, *p* < 0.01), magnesium (rho = −0.41, *p* < 0.05), vitamin D (rho = −0.59, *p* < 0.01), vitamin B6 (rho = −0.46, *p* < 0.01), niacin (rho = −0.44, *p* < 0.01), and vitamin B12 (rho = −0.32, *p* < 0.05). Additionally, significant inverse associations were observed between PR interval and zinc (rho = −0.38, *p* < 0.05), and between QRS and B12 (rho = 0.33, *p* < 0.05).

In the LA group, several inverse correlations were found between vitamin and mineral intake and cardiovascular parameters. Notably, QTc interval was negatively associated with zinc (rho = −0.62, *p* < 0.05), magnesium (rho = −0.64, *p* < 0.05), phosphorus (rho = −0.71, *p* < 0.05), selenium (rho = −0.85, *p* < 0.01), vitamin B12 (rho = −0.63, *p* < 0.05), and vitamin A (rho = −0.72, *p* < 0.01). Beta-carotene was inversely associated with PR interval (rho = −0.81, *p* < 0.01).

Additionally, systolic pressure showed strong inverse associations with zinc (rho = −0.82, *p* < 0.05), and cholesterol intake (rho = −0.82, *p* < 0.05), while diastolic pressure was negatively associated with carbohydrate intake (rho = −0.79, *p* < 0.05). Heart rate was inversely associated with vitamin C (rho = −0.61, *p* < 0.05).

## 4. Discussion

The primary objective of this study was to examine the potential impact of altitude exposure on cardiovascular and metabolic parameters in winter sports athletes, and to explore whether dietary intake could play a modulatory role. Based on previous evidence linking hypoxia to cardiac electrophysiology and metabolic stress alterations, we hypothesised that athletes training at higher altitudes would exhibit different electrocardiographic and biochemical profiles to those training at lower elevations. However, this study found no significant differences in ECG parameters, blood pressure or most metabolic markers between the HA and LA groups. These results suggest that an adequate nutritional profile may buffer the cardiovascular stress of hypoxia in these well-trained athletes [4].

The entire sample showed a favorable body composition profile, with a mean BMI of 21.02 kg/m^2^ and body fat percentage within expected ranges for athletic populations [35]. These values indicate that the participants were generally normal weight and metabolically healthy, reinforcing the interpretation of this cohort as a representative sample of health-preserving athletic performance.

Despite the absence of group differences in ECG parameters, several aspects warrant further consideration. The average resting heart rate in both groups (approximately 82 bpm) was slightly above the expected range for endurance-trained athletes (typically 66–72 bpm). This suggests either increased sympathetic tone or the influence of recent training load stimuli on the measurement [36]. Nevertheless, the PR intervals (144.0 ms), QRS durations (88.9 ms) and QT/QTc intervals (356.1 ms and 389.6 ms, respectively) were all within normal limits, confirming the absence of electrocardiographic abnormalities [1]. Similarly, both systolic and diastolic pressures remained within ranges considered normal, and even optimal, for physically active populations, highlighting the cardiovascular fitness of the sample. These findings echo previous studies on ski-mountaineering and mountaineering athletes, which reported preserved repolarisation despite hypoxia-induced sympathetic activation [5]. Resting systolic and diastolic pressures (≈107/63 mmHg) were within, or slightly below, the <120/80 mmHg range considered optimal for highly fit individuals. These results are consistent with those of other similar studies involving winter sports athletes [37], thus confirming the cardiovascular health of both groups.

The biochemical parameters were largely comparable between the groups, with one notable exception: the HA group had significantly higher lactate levels. This may be due to an increased reliance on anaerobic glycolysis caused by reduced oxygen availability (hypoxic stress), or it could be the result of higher levels of dehydration, which concentrate circulating metabolites [38]. Both mechanisms are plausible, as described in altitude physiology literature [39], and warrant further exploration.

Although current mean serum iron levels did not differ significantly between groups, the downward trend observed in the HA group suggests possible subclinical iron depletion, potentially linked to the well-documented effects of hypobaric hypoxia on iron metabolism [40]. Hypoxia-induced hepcidin upregulation may impair intestinal iron absorption and reduce iron recycling, a phenomenon observed in previous studies on athletes exposed to intermittent hypoxia [41]. Together, these findings justify longitudinal monitoring of lactate and iron status during altitude blocks and highlight the potential value of individualized iron-management strategies [10].

From a dietary perspective, although total energy intake, macronutrients intake, and overall caloric balance were similar between groups, differences in food source quality may explain variations in specific nutrient intake. Athletes in the HA group reported higher consumption of SFA, cholesterol, vitamin B_6_ and micronutrients typically derived from animal sources. In contrast, those in the LA group reported greater intake of glucose, β-carotene and vitamin C, suggesting a relatively more plant-based dietary pattern. According to the American Heart Association, primary dietary sources of SFA include meat and full-fat dairy products, whereas fruits and vegetables are the main contributors of antioxidants such as vitamin C and carotenoids [42]. The HA group’s preference for foods rich in SFA and heme iron may partly explain their higher circulating iron levels, despite comparable total iron intake. This is due to the significantly greater bioavailability of heme iron from animal sources compared to non-heme iron from plants [43]. The combination of these nutrient-dense foods could enhance iron absorption and overall nutritional resilience to hypoxic stress.

The correlation analysis supports our nutritional-modulation hypothesis. In HA athletes, higher intakes of magnesium, vitamins B_6_, B_12_ and D were associated with longer—but still normal—QT/QTc intervals and lower resting heart rate. Previous studies have linked magnesium and vitamin D to repolarisation dynamics and QT behaviour, while B vitamins participate in myocardial energy metabolism [44,45,46,47,48]. In LA athletes, greater vitamin C and β-carotene intake correlated with lower QTc and blood pressure, which is consistent with meta-analytic evidence suggesting that vitamin C supplementation can modestly reduce systolic and diastolic pressure [17,20,49]. Taken together, these findings suggest that it is micronutrient density rather than caloric amount that fine-tunes electrophysiological responses to training and the environment. It should be noted that these correlations reflect potential nutritional influences rather than established clinical effects, as most evidence relating vitamins D, C, and B-complex to cardiac repolarization has been reported in deficient or clinical populations rather than healthy, physically active individuals.

Earlier research documented transient QTc prolongation, rightward QRS axis deviation, and increased arrhythmic burden following rapid ascent to high altitude, particularly in non-acclimatized individuals [4,5]. In contrast, the athletes in the current study followed a “live low–train high” pattern throughout the ski season, regularly ascending to altitudes of 2500–3300 m for training sessions or multi-day camps, but residing at lower elevations. Although this exposure was intermittent rather than chronic, its cumulative nature—combined with nutritional support—may have facilitated partial physiological adaptation while limiting electrical instability. This model aligns with previous findings showing that repeated LL-TH exposures can induce beneficial cardiopulmonary and hematological adaptations when sustained over time [50]. Current findings thus extend previous observations by suggesting that even in the context of intermittent hypobaric hypoxia, well-planned dietary strategies, including adequate intake of electrolytes, B-complex vitamins, vitamin D, and antioxidants, could mitigate the pro-arrhythmic effects typically associated with high-altitude environments. Recent studies linking vitamin D deficiency to QTc prolongation further supported its potential role in maintaining repolarization stability under hypoxic stress [48].

### 4.1. Strengths and Limitations

The cross-sectional design of the current study prevents causal inference between altitude exposure, nutrient intake and cardiovascular outcomes. Dietary information relied on self-reported 24-h recalls that are prone to memory bias and systematic under-reporting in athletic populations. Additionally, basal metabolic rate was estimated through predictive equations rather than measured by indirect calorimetry, which may have introduced some degree of estimation error.

The unequal group distribution may have reduced the statistical power to detect small-to-moderate effects, although the study was sufficiently powered for moderate-to-large differences. Both altitude groups were homogeneous in age, sex distribution, and weekly training volume, minimizing potential confounding. However, other factors such as training modality, biological maturation, and hydration status could also have influenced the results. Minor non-significant differences in skinfold thickness and body fat percentage between groups were not statistically adjusted but could represent a potential source of residual confounding. As the correlation analyses were exploratory, the results should be interpreted with caution due to the increased risk of type I error associated with multiple testing.

### 4.2. Future Perspectives

Future investigations should corroborate dietary intake with objective biochemical markers and couple these with serial or continuous ECG monitoring across micro-cycles of training, in order to capture adaptation dynamics.

Looking ahead, longitudinal designs that follow athletes before, during and after altitude camps or that employ “live low–train high” models could clarify whether repeated exposure produces cumulative adaptations or reveals risk thresholds.

Promising avenues for future research include controlled nutritional interventions that manipulate key micronutrients, comparative analyses of whole dietary patterns (e.g., Mediterranean versus Western diets) and the integration of big data approaches or machine learning algorithms to detect real-time ECG signatures.

## 5. Conclusions

The current findings suggest that the winter sport athletes examined maintain an overall healthy cardiometabolic profile at both high altitude and low altitude. Even so, the combination of a mildly elevated resting heart rate and the higher lactate concentrations observed under hypoxic conditions points to some degree of physiological strain. Moreover, the correlations identified between key micronutrients (electrolytes, B-vitamins, vitamin D and antioxidants) and ventricular repolarisation parameters support the hypothesis that diet may influence the cardiac response to altitude-induced hypoxia.

Taken together, these observations highlight the potential value of a carefully tailored eating plan, adjusted for the demands of altitude, to help athletes to maintain electrical and metabolic cardiac stability throughout an entire competitive season. Nevertheless, these findings are associative in nature, and confirmation through longitudinal or interventional studies is required before drawing definitive conclusions.

## Figures and Tables

**Table 1:** Anthropometric characteristics of the study sample by altitude group.

Variable (Mean, SD)		Group		Sig.	Effect Size	
	Sample (N = 102)	High Altitude (n = 70)	Low Altitude (n = 32)	*p*	r	IC
Sex, females (n, %)	55 (57)	35 (50)	20 (62)	0.026	-	-
Age (years)	20.1 (4.2)	21.2 (6.0)	17.8 (8.6)	0.076	0.24	(0.00, 0.45)
Height (cm)	154.0 (19.1)	152.6 (21.6)	157.3 (11.5)	0.052	0.04	(0.01, 0.24)
Weight (kg)	52.1 (19.9)	49.9 (20.8)	57.2 (16.9)	0.175	0.01	(0.01, 0.36)
BMI (kg/m^2^)	21.0 (4.5)	20.3 (2.8)	22.6 (4.2)	0.252	0.07	(0.00, 0.42)
Tricipital skinfold (mm)	14.2 (6.1)	12.6 (4.5)	17.8 (4.2)	0.398	0.22	(0.00, 0.55)
Bicipital skinfold (mm)	8.4 (5.4)	7.4 (5.1)	10.4 (6.3)	0.334	0.15	(0.00, 0.50)
Subscapular skinfold (mm)	11.3 (6.5)	9.8 (5.4)	14.5 (5.4)	0.328	0.14	(0.10, 0.50)
Suprailiac skinfold (mm)	12.1 (10.3)	10.8 (10.9)	15.0 (7.6)	0.305	0.11	(0.01, 0.48)
Abdominal skinfold (mm)	15.6 (9.2)	14.6 (9.1)	17.9 (9.1)	0.203	0.02	(0.00, 0.40)
Thigh skinfold (mm)	21.1 (9.9)	19.6 (8.9)	24.3 (11.3)	0.240	0.05	(0.01, 0.44)
Midleg skinfold (mm)	14.5 (7.1)	12.5 (5.4)	18.9 (8.5)	0.385	0.20	(0.00, 0.55)
Faulkner Body Fat (%)	13.7 (4.2)	13.0 (3.8)	15.3 (4.7)	0.268	0.08	(0.02, 0.45)

**Table 2:** Electrocardiographic assessment and blood pressure by altitude group.

Variable (Mean, SD)		Group		Sig.	Effect Size	
	Sample	High Altitude	Low Altitude	*p*	r	IC
Heart rate (bpm)	82 (18)	82 (18)	82 (15)	0.757	0.01	(0.00, 0.35)
PR interval (ms)	144.0 (19.5)	145.6 (20.2)	137.9 (15.3)	0.141	0.40	(0.01, 0.43)
QRS duration (ms)	88.9 (13.8)	89.4 (14.6)	87.1 (10.4)	1.000	0.13	(0.00, 0.31)
QT interval (ms)	356.1 (33.8)	354.9 (34.4)	360.5 (32.4)	0.881	0.02	(0.00, 0.35)
QTc (ms)	389.6 (21.0)	388.2 (21.5)	394.8 (19.0)	0.555	0.08	(0.04, 0.32)
Diastolic pressure (mmHg)	63.3 (25.5)	72.4 (13.2)	73.1 (6.6)	0.885	0.02	(0.00, 0.31)
Systolic pressure (mmHg)	96.4 (37.9)	113.3 (19.0)	107.7 (10.0)	0.552	0.09	(0.07, 0.34)

**Table 3:** Blood Analysis by altitude groups.

		Group		Sig.	Effect Size	
Variable	Sample	High Altitude	Low Altitude	*p*	r	IC
Red Blood Cells, 10^6^/µL	5.1 (0.4)	5.1 (0.4)	5.1 (0.4)	0.664	0.04	(0.01, 0.23)
Hemoglobin, g/dL	14.5 (0.0)	14.5 (0.0)	14.5 (0.0)	0.376	0.09	(0.05, 0.27)
Hematocrit, %	42.1 (0.0)	42.1 (0.0)	42.1 (0.0)	0.376	0.09	(0.05, 0.32)
MCV, fL	90.2 (4.8)	90.2 (4.6)	90.3 (5.2)	0.965	0.03	(0.01, 0.24)
MCH, pg	30.3 (2.0)	30.1 (2.0)	30.7 (2.1)	0.209	0.12	(0.08, 0.32)
MCHC, g/dL	33.2 (1.6)	33.4 (1.56	32.8 (1.5)	0.122	0.15	(0.01, 0.36)
Glucose, mg/dL	91.2 (0.4)	91.1 (0.4)	91.3 (0.3)	0.133	0.15	(0.07, 0.33)
Creatinine, mg/dL	1.0 (0.0)	1.0 (0.0)	1.0 (0.0)	0.768	0.03	(0.01, 0.25)
Uric Acid, mg/dL	5.5 (0.0)	5.5 (0.0)	5.5 (0.0)	0.768	0.03	(0.01, 0.24)
Cholesterol, mg/dL	180.4 (0.1)	180.4 (0.1)	180.4 (0.2)	0.968	0.07	(0.04, 0.22)
HDL, mg/dL	54.9 (0.0)	54.9 (0.0)	54.9 (0.0)	0.968	0.04	(0.02, 0.22)
LDH, mg/dL	85.8 (6.0)	86.2 (5.7)	85.1 (6.4)	0.450	0.07	(0.04, 0.28)
Triglycerides, mg/dL	100.7 (0.2)	100.7 (0.2)	100.7 (0.2)	0.357	0.09	(0.02, 0.27)
Bilirubin, mg/dL	0.8 (0.2)	0.8 (0.2)	0.8 (0.2)	0.680	0.04	(0.01, 0.25)
Lactate, mg/dL	1.5 (0.5)	1.6 (0.5)	1.3 (0.5)	0.028	0.21	(0.03, 0.45)
Sodium, mEq/L	142.6 (1.2)	142.6 (1.2)	142.7 (1.2)	0.580	0.05	(0.04, 0.26)
Potassium, mEq/L	5.1 (0.0)	5.1 (0.0)	5.1 (0.0)	0.174	0.14	(0.07, 0.21)
Chloride, mEq/L	104.6 (2.9)	104.4 (2.8)	105.1 (2.9)	0.351	0.09	(0.05, 0.28)
Iron, µg/dL	90.8 (17.7)	88.6 (18.0)	95.8 (16.2)	0.057	0.18	(0.02, 0.37)

Note: MCV: Mean Corpuscular Volume; MCH: Mean Corpuscular Hemoglobin; MCHC: Mean Corpuscular Hemoglobin Concentration; HDL: High-Density Lipoprotein; LDH: Low-Density Lipoprotein.

**Table 4:** Macronutrients daily intake by altitude group.

		Group		Sig.	Effect Size	
Variable	Sample	High Altitude	Low Altitude	*p*	r	IC
TEE, kcal *	2069.3 (573.0)	2083.9 (663.7)	2036.1 (281.9)	0.613	0.08	(−0.03, 0.51)
Energy intake, kcal *	1808.0 (482.6)	1784.6 (489.7)	1861.0 (469.8)	0.460	−0.16	(−0.01, 0.27)
Water, g *	2631.4 (991.0)	2546.9 (963.3)	2822.03 (1041.6)	0.220	−0.28	(−0.15, 0.71)
Protein, g *	84.6 (29.9)	84.2 (28.5)	85.6 (33.3)	0.831	−0.05	(−0.04, 0.37)
Carbohydrate, g *	174.9 (57.1)	171.4 (58.4)	183.0 (54.0)	0.335	−0.20	(−0.00, 0.37)
Lipid, g *	80.3 (30.9)	79.7 (30.8)	81.4 (31.5)	0.801	−0.06	(−0.00, 0.27)
Simple carbohydrate						
Glucose, g *	7.8 (5.0)	7.1 (4.9)	9.5 (5.0)	0.024	0.51	(−0.08, 0.93)
Fructose, g *	10.3 (6.8)	9.8 (7.1)	11.5 (5.9)	0.232	−0.24	(−0.67, 0.18)
Lactose, g *	9.0 (6.7)	8.4 (6.3)	10.1 (7.4)	0.275	−0.25	(−0.68, 0.17)
Cholesterol, mg *	326.2 (207.7)	360.1 (214.4)	255.1 (175.4)	0.013	0.52	(0.08, 0.95)
Caloric profile						
Proteins, %	19.0 (6.7)	19.4 (7.4)	18.2 (4.6)	0.333	0.18	(−0.25, 0.61)
Carbohydrates, %	38.9 (8.2)	38.4 (8.0)	39.9 (8.5)	0.441	−0.17	(−0.59, 0.25)
Lipids, %	39.6 (7.5)	39.9 (7.5)	38.9 (7.6)	0.537	0.13	(−0.29, 0.56)
Lipids profile						
SFA, %	11.9 (4.2)	13.1 (3.7)	9.2 (4.0)	0.001	1.00	(0.58, 1.47)
MUFA, %	15.8 (5.7)	15.4 (6.3)	16.7 (3.9)	0.210	−0.23	(−0.65, 0.2)
PUFA, %	5.1 (2.3)	5.0 (2.5)	5.3 (1.8)	0.462	−0.14	(−0.56, 0.28)

Note: * mean (SD). TEE: total energy expenditure; SFA: saturated fatty acids; MUFA: Monounsaturated fatty acids; PUFA: Polyunsaturated fatty acids.

**Table 5:** Mineral and vitamin daily intake by altitude group.

		Group		Sig.	Effect Size	
Nutrient	Sample	High Altitude	Low Altitude	*p*	r	IC
Mineral						
Calcium, mg	772.1 (422.7)	806.8 (466.6)	699.1 (305.5)	0.339	0.09	(0.00, 0.33)
Iron, mg	13.7 (6.1)	13.9 (5.9)	13.2 (7.7)	0.264	0.11	(0.00, 0.32)
Iodine, µg	79.9 (39.4)	84.0 (37.4)	71.2 (42.5)	0.032	0.21	(0.04, 0.39)
Zinc, mg	9.2 (3.0)	9.5 (3.1)	8.7 (3.0)	0.363	0.09	(0.00, 0.29)
Magnesium, mg	257.3 (101.3)	275.0 (98.9)	220.2 (97.6)	0.003	0.30	(0.12, 0.49)
Sodium, mg	2682.8 (1387.4)	2872.2 (1523.0)	2285.7 (952.2)	0.113	0.16	(0.01, 0.33)
Potassium, mg	2628.4 (1002.3)	2827.7 (941.3)	2210.6 (1012.4)	0.001	0.33	(0.13, 0.51)
Manganese, mg	2.4 (1.3)	2.7 (1.5)	1.7 (0.6)	0.000	0.37	(0.21, 0.54)
Copper, mg	18.8 (65.6)	13.4 (37.7)	30.1 (102.0)	0.073	0.18	(0.01, 0.38)
Cobalt, mg	1.3 (0.7)	1.4 (0.5)	1.3 (0.9)	0.124	0.15	(0.00, 0.36)
Chromium, µg	41.2 (39.0)	43.3 (21.6)	36.9 (61.6)	0.001	0.34	(0.13, 0.52)
Phosphorus, mg	1275.2 (429.7)	1327.5 (406.2)	1165.3 (463.0)	0.044	0.20	(0.03, 0.41)
Chlorine, mg	1904.9 (831.0)	1923.9 (851.8)	1865.1 (797.8)	0.826	0.02	(0.00, 0.22)
Fluorine, mg	2.0 (1.0)	2.3 (1.0)	1.5 (0.9)	0.000	0.38	(0.21, 0.54)
Selenium, µg	96.1 (49.8)	99.9 (49.9)	87.8 (49.4)	0.256	0.11	(0.00, 0.31)
Vitamin						
Thiamine, mg	1.4 (0.7)	1.5 (0.7)	1.3 (0.8)	0.140	0.15	(0.00, 0.34)
Riboflavin, mg	1.7 (0.7)	1.64 (0.7)	1.7 (0.9)	0.567	0.05	(0.00, 0.26)
Niacin, mg	34.6 (12.4)	36.02(12.4)	31.7 (12.1)	0.133	0.15	(0.01, 0.35)
B6, mg	2.0 (0.9)	2.1 (0.9)	1.8 (0.9)	0.051	0.20	(0.02, 0.39)
Folic acid, µg	243.6 (133.5)	265.69 (137.4)	197.4 (13.76)	0.018	0.24	(0.05, 0.44)
B_12_, µg	5.0 (5.2)	5.2 (6.1)	4.8 (2.93)	0.573	0.05	(0.00, 0.26)
Vitamin C, mg	95.7 (81.5)	105.1 (88.9)	75.9 (59.9)	0.064	0.19	(0.02, 0.39)
Vitamin A, µg	775.5 (537.7)	811.6 (506.4)	699.8 (599.8)	0.101	0.16	(0.02, 0.36)
Retinol, µg	373.8 (343.7)	365.43(325.2)	391.3 (384.8)	0.928	0.01	(0.03, 0.24)
Beta-carotene, µg	1910.3 (1866.4)	2144.3 (1885.4)	1419.7 (1754.8)	0.027	0.22	(0.03, 0.42)
Vitamin D, µg	3.2 (4.3)	2.7 (2.8)	4.3 (6.3)	0.925	0.01	(0.00, 0.25)
Vitamin E, mg	7.6 (4.5)	8.4 (5.0)	6.1 (2.6)	0.036	0.21	(0.02, 0.39)
Vitamin K, µg	143.9 (158.3)	144.4 (130.5)	142.7 (207.4)	0.061	0.19	(0.01, 0.44)

**Table 6:** Correlation between blood pressure and ECG with nutritional parameters (HA group).

Nutrient	Heart Rate	PR	QRS	QT	QTc	Diastolic Pressure	Systolic Pressure
TEE, kcal	−0.46 **	0.11	0.52 ***	0.56 ***	0.35 *	0.27	0.55 **
Energy intake, kcal	0.04	0.1	0.04	−0.17	−0.27	0.06	0.24
Macronutrients							
Water	0.23	0.18	−0.09	−0.16	−0.16	0.1	0.14
Protein	−0.14	0.15	0	0.02	−0.1	0.01	0.07
Carbohydrate	−0.07	0.04	0.11	−0.01	−0.15	0	0.11
Lipid	0.13	0.14	0.04	−0.22	−0.26	0.17	0.44 **
Glucose	0.12	0.1	0.04	0.05	0.13	0.16	0.09
Fructose	0.22	−0.02	−0.05	−0.05	0.1	−0.01	−0.09
Lactose	0.18	0.04	−0.17	−0.2	−0.11	−0.05	0.17
Cholesterol	0.03	−0.31	0.2	0.18	0.21	0.11	0.05
Mineral							
Calcium	0.01	−0.11	−0.15	−0.07	−0.03	−0.27	−0.19
Iron	−0.59 **	−0.17	0.15	0.42 **	0.26	−0.16	−0.19
Iodine	−0.32	−0.06	−0.02	0.25	0.32	−0.45 *	−0.17
Zinc	−0.33 *	−0.38 *	−0.01	0.35 *	0.22	−0.04	−0.16
Magnesium	−0.41 *	−0.15	0.00	0.33 *	0.28	−0.18	0.06
Sodium	−0.19	−0.02	0.02	0.15	0.13	−0.07	−0.02
Potassium	−0.13	−0.14	−0.21	−0.12	−0.25	−0.12	−0.35
Manganese	−0.45 *	−0.14	−0.06	0.26	0.17	−0.26	0.09
Copper	−0.51 **	0.03	−0.11	0.24	0.04	−0.15	−0.17
Cobalt	−0.21	−0.22	−0.21	−0.06	−0.22	−0.33	−0.48 **
Chromium	0.16	0.03	−0.17	−0.14	−0.05	−0.04	−0.26
Chlorine	−0.18	−0.05	0.14	0.05	−0.02	0.02	−0.07
Fluorine	−0.14	−0.23	−0.07	0.19	−0.13	0.10	−0.01
Phosphorus	−0.37 *	−0.14	−0.08	0.31	0.23	−0.21	−0.08
Selenium	−0.22	0.03	0.00	0.22	0.25	−0.12	0.04
Vitamins							
Thiamine	−0.15	−0.13	−0.1	0.18	0.02	−0.21	−0.34
Riboflavin	−0.29	−0.09	−0.04	0.27	0.1	−0.19	−0.25
Niacin	−0.44 **	−0.15	0.12	0.47 **	0.34 *	0.12	0.21
B6	−0.46 **	0.12	0.02	0.45 **	0.18	0.03	0.05
Folic acid	−0.24	0.23	0.07	0.07	−0.05	0.02	0.05
B12	−0.32 *	−0.29	0.33 *	0.46 **	0.39 *	−0.01	0.09
Vitamin C	−0.29	0.19	−0.14	0.06	−0.22	−0.03	−0.09
Vitamin A	0.01	0.19	0.13	0.00	0.11	−0.13	−0.24
Vitamin D	−0.59 **	0.12	0.28	0.42 **	0.12	−0.08	0.04
Vitamin E	−0.08	0.09	−0.06	0.09	0.14	0.11	0.24
Vitamin K	−0.22	−0.03	0.23	0.14	0.17	−0.13	−0.14
Retinol	−0.19	0.08	0.22	0.23	0.14	−0.07	−0.17
Beta-carotene	0.12	0.13	−0.03	−0.12	0.04	−0.11	−0.16

Note: * *p* ≤ 0.050; ** *p* ≤ 0.010; *** *p* ≤ 0.001.

**Table 7:** Correlation between blood pressure and ECG with nutritional parameters (LA group).

Nutrient	Heart Rate	PR	QRS	QT	QTc	Diastolic Pressure	Systolic Pressure
Macronutrients							
TEE, kcal	−0.39	0.12	0.36	0.56	0.55	0.07	0.71
Energy intake, kcal	−0.02	−0.13	0.27	0.13	0.27	−0.14	0.11
Water	0.23	0.29	−0.15	0.02	0.19	0.36	0.82 *
Protein	0.08	−0.07	0.07	0.02	0.16	−0.04	0.32
Carbohydrate	0.15	0.24	0.08	−0.29	−0.25	−0.79 *	−0.75
Lipid	0.03	−0.16	0.09	0.15	0.23	0.14	0.18
Glucose	0.55	−0.37	−0.48	−0.61 *	−0.4	−0.32	0.07
Fructose	0.57	−0.34	−0.45	−0.57	−0.31	−0.32	0.07
Lactose	0.18	0.01	0.05	0.14	0.31	0.47	0.24
Cholesterol	0.06	−0.12	0.26	0.09	−0.01	0.00	−0.82 *
Mineral							
Calcium	0.18	−0.53	0.00	−0.38	−0.54	−0.32	−0.46
Iron	0.42	−0.31	−0.14	−0.39	−0.48	0.29	−0.39
Iodine	0.08	−0.24	0.21	−0.39	−0.59 *	−0.36	−0.57
Zinc	0.27	−0.34	0.01	−0.50	−0.62 *	−0.14	−0.82 *
Magnesium	0.26	−0.66 *	−0.09	−0.61 *	−0.64 *	−0.11	−0.5
Sodium	−0.12	−0.51	0.51	−0.13	−0.27	−0.21	−0.5
Potassium	0.15	−0.57	0.13	−0.42	−0.51	0.07	−0.43
Manganese	0.11	−0.74 *	0.17	−0.21	−0.01	−0.31	0
Copper	0.13	−0.77 **	−0.06	−0.37	−0.37	−0.07	−0.14
Cobalt	0.51	−0.62 *	−0.28	−0.61 *	−0.55	−0.46	−0.36
Chromium	−0.18	−0.63 *	0.25	−0.09	−0.27	−0.21	−0.18
Chlorine	0.08	−0.48	0.16	−0.30	−0.49	0.14	−0.32
Fluorine	0.02	−0.39	0.12	−0.16	−0.34	0.07	0.11
Phosphorus	0.56	−0.33	−0.18	−0.64 *	−0.71 *	−0.07	−0.36
Selenium	0.44	−0.44	−0.19	−0.69 *	−0.85 **	−0.61	−0.75
Vitamin							
Thiamine	−0.13	−0.10	0.40	0.18	0.00	0.57	−0.04
Riboflavin	0.28	0.08	−0.28	−0.43	−0.54	−0.04	−0.63
Niacin	0.42	−0.15	0.11	−0.35	−0.41	0.21	−0.57
B6	0.10	−0.03	0.28	−0.01	−0.16	0.39	−0.14
Folic acid	−0.08	−0.27	0.32	−0.01	−0.22	0.29	−0.21
B12	0.37	−0.08	−0.01	−0.51	−0.63 *	−0.29	−0.46
Vitamin C	−0.61 *	−0.02	0.53	0.50	0.10	0.09	−0.23
Vitamin A	0.24	−0.72 *	0.12	−0.35	−0.36	−0.25	−0.43
Vitamin D	0.35	−0.03	0.13	−0.2	−0.18	0.39	−0.14
Vitamin E	−0.03	−0.23	0.25	−0.35	−0.49	0.14	−0.61
Vitamin K	−0.25	−0.15	0.00	−0.23	−0.31	−0.21	−0.5
Retinol	0.55	−0.32	−0.19	−0.44	−0.41	−0.25	−0.43
Beta-carotene	−0.10	−0.81 **	0.25	−0.24	−0.33	−0.21	−0.71

Note: * *p* ≤ 0.050; ** *p* ≤ 0.010.

## Data Availability

There are restrictions on the availability of data for this trial due to the signed consent agreements around data sharing, which only allow access to external researchers for studies following the project’s purposes. Requestors wishing to access the trial data used in this study can make a request to mariscal@ugr.es.

## References

[1] Sharma S., Drezner J.A., Baggish A., Papadakis M., Wilson M.G., Prutkin J.M., La Gerche A., Ackerman M.J., Börjesson M., Salerno J.C. (2018). International recommendations for electrocardiographic interpretation in athletes. Eur. Heart J..

[2] Basu J., Malhotra A. (2018). Interpreting the Athlete’s ECG: Current State and Future Perspectives. Curr. Treat. Options Cardiovasc. Med..

[3] Parodi J.B., Ramchandani R., Zhou Z., Chango D.X., Acunzo R., Liblik K., Farina J.M., Zaidel E.J., Ruiz-Mori E., Carreón J.M.A. (2023). A systematic review of electrocardiographic changes in healthy high-altitude populations. Trends Cardiovasc. Med..

[4] Ramchandani R., Zhou Z., Parodi J.B., Farina J.M., Liblik K., Sotomayor J., Burak C., Herman R., Baranchuk A. (2023). A Systematic Review of Electrocardiographic Changes in Populations Temporarily Ascending to High Altitudes. Curr. Probl. Cardiol..

[5] Coustet B., Lhuissier F.J., Vincent R., Richalet J.P. (2015). Electrocardiographic Changes During Exercise in Acute Hypoxia and Susceptibility to Severe High-Altitude Illnesses. Circulation.

[6] Gudelunas K., Chinn G.A., Barreto-Chang O.L., Campbell L., Sall J.W. (2025). A 4-Day Exposure to High Altitude Prolongs QTc in Healthy Human Subjects. Wilderness Environ. Med..

[7] Zimmermann P., Eckstein M.L., Moser O., Schöffl I., Zimmermann L., Schöffl V. (2022). Left Ventricular, Left Atrial and Right Ventricular Strain Modifications after Maximal Exercise in Elite Ski-Mountaineering Athletes: A Feasibility Speckle Tracking Study. Int. J. Environ. Res. Public Health.

[8] Carta A.F., Bitos K., Furian M., Mademilov M., Sheraliev U., Marazhapov N.H., Lichtblau M., Schneider S.R., Sooronbaev T., Bloch K.E. (2021). ECG changes at rest and during exercise in lowlanders with COPD travelling to 3100 m. Int. J. Cardiol..

[9] Shah A.B., Coplan N. (2016). Cardiovascular Effects of Altitude on Performance Athletes. Rev. Cardiovasc. Med..

[10] Girard O., Levine B.D., Chapman R.F., Wilber R. (2023). “Living High-Training Low” for Olympic Medal Performance: What Have We Learned 25 Years After Implementation?. Int. J. Sports Physiol. Perform..

[11] Conde-Pipó J., Valenzuela-Barranco I., López-Moro A., Román-Alconchel B., Mariscal-Arcas M., Zurita-Ortega F. (2022). Influence of Alpine Skiing on Health-Related Quality of Life and Physical Self-Concept in Physically Active Adults over 55 Years of Age. Sports..

[12] Muller E., Gimpl M., Poetzelsberger B., Finkenzeller T., Scheiber P. (2011). Salzburg Skiing for the Elderly Study: Study design and intervention—Health benefit of alpine skiing for elderly. Scand. J. Med. Sci. Sports.

[13] Conde-Pipó J., Valenzuela-Barranco I., Peñafiel R., Jiménez-Casquet M.J., López-Moro A., Mariscal-Arcas M. (2022). Vital parameters in Spanish alpine skiers training at altitude and their relationship to human health. Arch. Nurs. Pract. Care.

[14] Niederseer D., Steidle-Kloc E., Mayr M., Müller E.E., Cadamuro J., Patsch W., Dela F., Müller E., Niebauer J. (2016). Effects of a 12-week alpine skiing intervention on endothelial progenitor cells, peripheral arterial tone and endothelial biomarkers in the elderly. Int. J. Cardiol..

[15] Krautgasser S., Scheiber P., von Duvillard S.P., Müller E. (2011). Physiological responses of elderly recreational alpine skiers of different fitness and skiing abilities. J. Sports Sci. Med..

[16] Rossi V.A., Schmied C., Niebauer J., Niederseer D. (2019). Cardiovascular effects and risks of recreational alpine skiing in the elderly. J. Sci. Med. Sport.

[17] D’Imperio S., Monasky M.M., Micaglio E., Negro G., Pappone C. (2021). Impact of Dietary Factors on Brugada Syndrome and Long QT Syndrome. Nutrients..

[18] Strüven A., Holzapfel C., Stremmel C., Brunner S. (2021). Obesity, Nutrition and Heart Rate Variability. Int. J. Mol. Sci..

[19] Marstrand P., Almatlouh K., Kanters J.K., Graff C., Christensen A.H., Bundgaard H., Theilade J. (2021). Effect of moderate potassium-elevating treatment in long QT syndrome: The TriQarr Potassium Study. Open Heart.

[20] Chen Y., Guo X., Sun G., Li Z., Zheng L., Sun Y. (2018). Effect of serum electrolytes within normal ranges on QTc prolongation: A cross-sectional study in a Chinese rural general population. BMC Cardiovasc. Disord..

[21] Etheridge S.P., Compton S.J., Tristani-Firouzi M., Mason J.W. (2003). A new oral therapy for long QT syndrome. J. Am. Coll. Cardiol..

[22] Hannon M.P., Flueck J.L., Gremeaux V., Place N., Kayser B., Donnelly C. (2021). Key Nutritional Considerations for Youth Winter Sports Athletes to Optimize Growth, Maturation and Sporting Development. Front. Sports Act. Living.

[23] Meyer N.L., Manore M.M., Helle C. (2011). Nutrition for winter sports. J. Sports Sci..

[24] Jiménez-Casquet M.J., Conde-Pipó J., Valenzuela-Barranco I., Rienda-Contreras R., Olea-Serrano F., Bouzas C., Tur J.A., Mariscal-Arcas M. (2023). Nutrition Status of Female Winter Sports Athletes. Nutrients.

[25] Franco M.V., Giménez-Blasi N., Latorre J.A., Martínez-Bebia M., Bach A., Olea-Serrano F., Mariscal-Arcas M. (2020). Actualización sobre deficiencias nutricionales en la mujer deportista a partir de la literatura científica. Arch. Latinoam. Nutr..

[26] Stellingwerff T., Peeling P., Garvican-Lewis L.A., Hall R., Koivisto A.E., Heikura I.A., Burke L.M. (2019). Nutrition and Altitude: Strategies to Enhance Adaptation, Improve Performance and Maintain Health: A Narrative Review. Sports Med..

[27] Papadimitriou K., Detopoulou P., Soufleris K., Voulgaridou G., Tsoumana D., Ntopromireskou P., Giaginis C., Chatziprodromidou I.P., Spanoudaki M., Papadopoulou S.K. (2023). Nutritional Risk and Sarcopenia Features in Patients with Crohn’s Disease: Relation to Body Composition, Physical Performance, Nutritional Questionnaires and Biomarkers. Nutrients.

[28] Conde-Pipó J., Bouzas C., Mariscal-Arcas M., Tur J.A. (2022). Association between Functional Fitness and Health-Related Quality of Life in the Balearic Islands’ Old Adults with Metabolic Syndrome. Nutrients.

[29] Martínez-Rodríguez A., Sánchez-Sánchez J., Vicente-Martínez M., Martínez-Olcina M., Miralles-Amorós L., Sánchez-Sáez J.A. (2021). Anthropometric dimensions and bone quality in international male beach handball players: Junior vs. senior comparison. Nutrients.

[30] Kerksick C.M., Kulovitz M. (2013). Requirements of Energy, Carbohydrates, Proteins and Fats for Athletes. Nutrition and Enhanced Sports Performance.

[31] World Health Organization (1985). Energy and Protein Requirements: Report of a Joint FAO/WHO/UNU Expert Consultation.

[32] Rivas A., Romero A., Mariscal M., Monteagudo C., Hernández J., Olea-Serrano F. (2009). Validation of questionnaires for the study of food habits and bone mass. Nutr. Hosp..

[33] Goldberg G.R., Black A.E. (1998). Assessment of the validity of reported energy intakes—Review and recent developments. Näringsforskning.

[34] Black A. (2000). Critical evaluation of energy intake using the Goldberg cut-off for energy intake: Basal metabolic rate. A practical guide to its calculation, use and limitations. Int. J. Obes..

[35] Garrido-Chamorro R.P., Sirvent-Belando J.E., Gonzalez-Lorenzo M., Martin-Carratala M.L., Roche E. (2009). Correlation between body mass index and body composition in elite athletes. J. Sports Med. Phys. Fit..

[36] Auza-Santivañez J.C., Quisbert Vasquez H.T., Bautista-Vanegas F.E., Espejo-Alanoca D., Chiri-Chambi P., Mamani Huarachi V.H., Aguirre-Cruz B., Sivila-Marquez K.D., Diaz-Guerrero J.L. (2025). Impact of Altitude on Cardiovascular Physiology: Literature Review and Update. Health Leadersh. Qual. Life.

[37] Zimmermann P., Moser O., Eckstein M.L., Wüstenfeld J., Schöffl V., Zimmermann L., Braun M., Schöffl I. (2021). Athlete’s Heart in Elite Biathlon, Nordic Cross—Country and Ski-Mountaineering Athletes: Cardiac Adaptions Determined Using Echocardiographic Data. J. Cardiovasc. Dev. Dis..

[38] Burtscher M., Álvarez-Herms J., Burtscher J., Strasser B., Kopp M., Pageaux B. (2025). Could the perception of effort help us unravel the potential of “living low–training high”? A perspective article. J. Sports Sci..

[39] West J.B. (2007). Point: The lactate paradox does/does not occur during exercise at high altitude. J. Appl. Physiol..

[40] Karpęcka-Gałka E., Frączek B. (2024). Nutrition, hydration and supplementation considerations for mountaineers in high-altitude conditions: A narrative review. Front. Sports Act. Living.

[41] Badenhorst C.E., Dawson B., Goodman C., Sim M., Cox G.R., Gore C.J., Tjalsma H., Swinkels D.W., Peeling P. (2014). Influence of post-exercise hypoxic exposure on hepcidin response in athletes. Eur. J. Appl. Physiol..

[42] Sacks F.M., Lichtenstein A.H., Wu J.H.Y., Appel L.J., Creager M.A., Kris-Etherton P.M., Miller M., Rimm E.B., Rudel L.L., Robinson J.G. (2017). Dietary Fats and Cardiovascular Disease: A Presidential Advisory From the American Heart Association. Circulation.

[43] Sim M., Garvican-Lewis L.A., Cox G.R., Govus A., McKay A.K., Stellingwerff T., Peeling P. (2019). Iron considerations for the athlete: A narrative review. Eur. J. Appl. Physiol..

[44] Dinicolantonio J.J., Liu J., O’Keefe J.H. (2018). Magnesium for the prevention and treatment of cardiovascular disease. Open Heart.

[45] Nielsen F.H. (2024). The Role of Dietary Magnesium in Cardiovascular Disease. Nutrients.

[46] Rosique-Esteban N., Guasch-Ferré M., Hernández-Alonso P., Salas-Salvadó J. (2018). Dietary Magnesium and Cardiovascular Disease: A Review with Emphasis in Epidemiological Studies. Nutrients.

[47] Strohm D., Bechthold A., Isik N., Leschik-Bonnet E., Heseker H. (2016). Revised reference values for the intake of thiamin (vitamin B1), riboflavin (vitamin B2), and niacin. NFS J..

[48] Bagrul D., Atik F. (2019). Association of vitamin D deficiency with ventricular repolarization abnormalities. Kardiol. Pol..

[49] Juraschek S.P., Guallar E., Appel L.J., Miller E.R. (2012). Effects of vitamin C supplementation on blood pressure: A meta-analysis of randomized controlled trials. Am. J. Clin. Nutr..

[50] Chapman R.F., Karlsen T., Resaland G.K., Ge R.-L., Harber M.P., Witkowski S., Stray-Gundersen J., Levine B.D. (2014). Defining the “dose” of altitude training: How high to live for optimal sea level performance enhancement. J. Appl. Physiol..

